# Leveraging neuroinformatics to understand cognitive phenotypes in elite athletes through systems neuroscience

**DOI:** 10.3389/fninf.2025.1557879

**Published:** 2025-08-19

**Authors:** Yubin Huang, Jun Liu, Qi Yu

**Affiliations:** ^1^Department of Rehabilitation Medicine, Ganzhou People's Hospital, Ganzhou, China; ^2^Ganzhou People's Hospital, Ganzhou, China; ^3^College of Art, Shaanxi University of Technology, Hanzhong, Shaanxi, China

**Keywords:** neuroinformatics, cognitive phenotypes, elite athletes, systems neuroscience, deep learning

## Abstract

**Introduction:**

Understanding the cognitive phenotypes of elite athletes offers a unique perspective on the intricate interplay between neurological traits and high-performance behaviors. This study aligns with advancing neuroinformatics by proposing a novel framework designed to capture and analyze the multi-dimensional dependencies of cognitive phenotypes using systems neuroscience methodologies. Traditional approaches often face limitations in disentangling the latent factors influencing cognitive variability or in preserving interpretable data structures.

**Methods:**

To address these challenges, we developed the Latent Cognitive Embedding Network (LCEN), an innovative model that combines biologically inspired constraints with state-of-the-art neural architectures. The model features a specialized embedding mechanism for disentangling latent factors and a tailored optimization strategy incorporating domain-specific priors and regularization techniques.

**Results:**

Experimental evaluations demonstrate LCEN's superiority in predicting and interpreting cognitive phenotypes across diverse datasets, providing deeper insights into the neural underpinnings of elite performance.

**Discussion:**

This work bridges computational modeling, neuroscience, and psychology, contributing to the broader understanding of cognitive variability in specialized populations.

## 1 Introduction

Understanding cognitive phenotypes in elite athletes is essential for unraveling the neural mechanisms that underlie exceptional performance (Hu et al., 2023). These phenotypes, which include heightened attention, faster reaction times, and superior decision-making, are not only critical for advancing sports science but also provide valuable insights into broader neurocognitive processes (Wei et al., 2023). The integration of neuroinformatics and systems neuroscience enables researchers to analyze complex data streams and model the interplay between brain networks, offering a comprehensive framework for studying these elite cognitive traits (Wang et al., 2023). By applying neuroinformatics approaches, it is not only possible to identify biomarkers of elite cognitive function but also to explore how neural adaptations are influenced by intense training. This area of research holds potential for applications in enhancing athletic performance, understanding brain plasticity, and even informing clinical interventions for cognitive enhancement (Zong et al., 2023). To address the limitations of traditional methods in studying cognitive phenotypes, researchers initially relied on symbolic AI and knowledge-based approaches. These methods focused on structured data representations and rule-based systems to interpret cognitive traits, leveraging well-established neuroscience theories and statistical models (Xu et al., 2023). Symbolic approaches were particularly effective in understanding specific aspects of cognition, such as decision-making and attention control, through frameworks like expert systems and neurocognitive modeling. However, these methods struggled with scalability and the integration of multimodal data, such as imaging and behavioral datasets (Peng et al., 2022). Moreover, they lacked the capacity to account for dynamic neural processes and individual variability, which are critical for understanding elite athletes' unique cognitive adaptations.

As neuroinformatics advanced, data-driven and machine learning approaches began to play a central role in analyzing cognitive phenotypes (Xu et al., 2022). These techniques enabled the extraction of patterns from large datasets, such as functional MRI, EEG, and behavioral measures, to predict and characterize elite cognitive performance. Methods like clustering, classification, and regression provided new insights into brain-behavior relationships, while models such as support vector machines (SVM) and random forests helped identify key features associated with superior cognitive function (Song et al., 2023). Despite these advancements, traditional machine learning approaches often required extensive preprocessing and feature engineering, limiting their flexibility. They struggled with generalizability across diverse athlete populations and failed to capture the complexity of dynamic neural networks involved in high-performance cognition (Yao et al., 2023). The emergence of deep learning and pre-trained neural network models has revolutionized the study of cognitive phenotypes in elite athletes. These methods excel at processing multimodal and high-dimensional data, such as combining neuroimaging, genetics, and behavioral measures (Zhou H.-Y. et al., 2023). Models like convolutional neural networks (CNNs) and transformers have been applied to identify neural signatures of elite performance, while pre-trained models, such as BERT and GPT, have shown promise in decoding cognitive traits from textual and symbolic data sources (Zhang et al., 2023). These approaches address many limitations of earlier methods by enabling end-to-end learning and capturing temporal dynamics within neural systems (Shi et al., 2022). However, deep learning models often require large-scale datasets and substantial computational resources, posing challenges for studies with limited sample sizes (Hao et al., 2022). Furthermore, the interpretability of these models remains a key limitation, as understanding the neural mechanisms underlying elite performance is as critical as achieving accurate predictions (Joseph et al., 2023).

Building upon the limitations of traditional symbolic approaches, machine learning, and deep learning methods, this study proposes a novel framework for leveraging neuroinformatics to analyze cognitive phenotypes in elite athletes (Zhang et al., 2022). Our approach integrates advanced systems neuroscience models with a multi-scale neuroinformatics pipeline, combining functional, structural, and behavioral data to overcome the limitations of previous methods. By incorporating explainable AI techniques and domain-specific models, our framework aims to improve both the interpretability and generalizability of findings. We propose a modular design that can be adapted for diverse athlete populations and cognitive domains, addressing key challenges in scalability and data integration.

The proposed method has several key advantages:

- The proposed framework introduces a hybrid approach combining explainable AI and multi-scale neuroinformatics, providing a novel way to analyze cognitive phenotypes in elite athletes.
- Our method is designed to be scalable and adaptable across multiple contexts, including different sports and cognitive tasks, enhancing its applicability and efficiency.
- Preliminary findings demonstrate that our framework achieves superior accuracy and interpretability compared to existing methods, identifying novel biomarkers of elite cognitive performance.

## 2 Related work

### 2.1 Neuroinformatics in cognitive profiling

The integration of neuroinformatics into cognitive research has significantly advanced our understanding of cognitive phenotypes by enabling the aggregation, analysis, and modeling of complex neurobiological datasets (Silverio and Silverio, 2022). Neuroinformatics leverages computational techniques to manage the vast quantities of data generated through neuroimaging modalities such as functional magnetic resonance imaging (fMRI), magnetoencephalography (MEG), and diffusion tensor imaging (DTI). This approach is particularly relevant to studying elite athletes, where precise cognitive profiling requires the synthesis of neural, genetic, and behavioral data into actionable insights. A major focus of neuroinformatics in cognitive profiling is the identification of neurobiological correlates of high performance, such as enhanced motor planning, decision-making, and situational awareness (Lian et al., 2022). By utilizing data-driven models, researchers can map patterns of brain connectivity and activation associated with these traits. For instance, graph theoretical approaches applied to functional connectivity networks have revealed the role of modularity and hub regions such as the dorsolateral prefrontal cortex in supporting rapid decision-making. These insights are especially pertinent to elite athletes, whose exceptional cognitive abilities often depend on efficient neural network organization (Liu et al., 2023). Neuroinformatics frameworks also facilitate the integration of multimodal datasets, combining structural and functional imaging with electroencephalography (EEG) measures to provide a more comprehensive picture of cognitive phenotypes. Machine learning algorithms, a key component of neuroinformatics, are increasingly employed to classify cognitive phenotypes based on neural features. In elite athletes, such algorithms have been used to distinguish between individuals with varying levels of expertise in sports requiring rapid response and adaptability (Steyaert et al., 2023). Predictive models trained on neuroimaging data have shown promise in identifying key features that distinguish elite performers, such as increased connectivity within sensorimotor networks and heightened activity in the anterior cingulate cortex. Moreover, neuroinformatics tools allow for the longitudinal analysis of cognitive phenotypes, enabling researchers to study how these traits develop over time and in response to training interventions (Du et al., 2022). Another critical aspect of neuroinformatics is its role in managing the inherent variability of neural data. Elite athletes often exhibit unique neural adaptations that may not conform to general population norms. Neuroinformatics systems address this challenge through individualized modeling approaches that account for inter-individual differences. For example, personalized connectome analyses have highlighted variations in motor planning networks that correlate with specific sports disciplines. Such tailored analyses not only improve the accuracy of cognitive profiling but also provide insights into the neural basis of specialized skills. The application of neuroinformatics in cognitive profiling extends beyond analysis to include visualization and hypothesis generation. Advanced visualization techniques, such as connectome mapping and brain atlases, enable researchers to intuitively explore the relationships between brain structure and cognitive function. These tools are invaluable for generating hypotheses about the neural mechanisms underlying elite performance, which can then be tested through targeted experiments. The use of computational models to simulate neural processes provides a framework for understanding how specific neural adaptations contribute to cognitive phenotypes. In the context of elite athletes, neuroinformatics also has implications for training and performance optimization. By identifying the neural correlates of cognitive strengths and weaknesses, researchers can develop targeted interventions to enhance performance. For instance, neurofeedback and brain stimulation techniques informed by neuroinformatics analyses have shown potential in improving attention and motor control. Such applications highlight the transformative potential of neuroinformatics in both research and practice.

While recent advances in multimodal learning have enabled novel approaches to modeling brain-behavior relationships, it is crucial to ground these computational techniques within the domain-specific context of sports neuroscience. Previous studies have established standardized cognitive profiling protocols tailored to elite athlete populations, including reaction-time benchmarks, attention control tasks, and situational decision-making assessments in ecologically valid settings (Vestberg et al., 2012). These domain-specific traits often differ markedly from general population baselines, necessitating discipline-sensitive modeling strategies. Moreover, experimental designs in sports science frequently incorporate specialized intervention protocols and require careful power analysis due to cohort limitations (Mirifar et al., 2019). Studies such as “Neurocognitive Profiling in Elite Performers” highlight how training regimens and task design influence measurable cognitive traits, offering critical insights for embedding ecological validity into neuroinformatics pipelines. To ensure methodological rigor, we draw on these works to refine phenotype selection and justify the statistical robustness of our athlete-specific datasets (Eckner et al., 2010).

### 2.2 Systems neuroscience in athletic cognition

Systems neuroscience provides a comprehensive framework for understanding the neural mechanisms underlying cognitive performance, particularly in elite athletes who exhibit extraordinary capabilities in domains such as attention, decision-making, and motor control (Raufi and Longo, 2022). By examining the interactions between neural circuits and their contributions to cognitive processes, systems neuroscience offers critical insights into the neural adaptations that support high-level performance. One of the primary areas of interest in systems neuroscience is the role of large-scale brain networks in cognitive function (Chai and Wang, 2022). The default mode network (DMN), central executive network (CEN), and salience network (SN) have been implicated in various aspects of cognitive performance relevant to elite athletes. For example, the DMN's deactivation during goal-directed tasks allows for enhanced focus and situational awareness, while the CEN supports complex decision-making and working memory (Zhou Y. et al., 2023). The SN, on the other hand, facilitates the dynamic switching between these networks, enabling athletes to rapidly adapt to changing circumstances. Studies employing fMRI and MEG have demonstrated that elite athletes exhibit enhanced functional connectivity within these networks, which correlates with superior cognitive performance (Lin et al., 2023). Systems neuroscience also emphasizes the importance of sensorimotor integration in athletic cognition. The ability to seamlessly integrate sensory inputs with motor outputs is a hallmark of elite performance, particularly in sports requiring split-second decisions and precise movements (Yan et al., 2022). Research has shown that the cerebellum, basal ganglia, and primary motor cortex play pivotal roles in this process, with enhanced connectivity between these regions observed in elite athletes (Fan et al., 2022). Moreover, the prefrontal cortex contributes to the top-down modulation of motor responses, ensuring that actions are contextually appropriate and aligned with performance goals. Plasticity is another key concept in systems neuroscience that is highly relevant to understanding athletic cognition. Neuroplasticity refers to the brain's ability to adapt structurally and functionally in response to training and experience. In elite athletes, intensive practice leads to region-specific plasticity, such as increased gray matter volume in motor and visuospatial regions. Longitudinal studies have further revealed that these adaptations are not static but continue to evolve with ongoing training. This dynamic nature of neural plasticity underscores the importance of systems neuroscience in capturing the temporal aspects of cognitive and neural changes in athletes. Techniques such as optogenetics and transcranial magnetic stimulation (TMS) have allowed researchers to directly manipulate neural activity within specific circuits, providing causal evidence for their roles in athletic cognition. For instance, stimulation of the pre-motor cortex has been shown to enhance motor planning and execution, while inhibition of the anterior cingulate cortex impairs error monitoring and correction. These findings highlight the potential of systems neuroscience not only to elucidate the neural mechanisms underlying athletic performance but also to inform the development of targeted interventions. The interplay between systems neuroscience and cognitive phenotypes is further exemplified by its application in understanding the effects of fatigue and stress on performance. Neural circuits involved in attention and decision-making are particularly susceptible to the detrimental effects of these factors, which can compromise performance even in highly trained individuals. Systems neuroscience approaches, including computational modeling and network analysis, have been instrumental in identifying the neural correlates of fatigue and developing strategies to mitigate its impact.

### 2.3 Cognitive phenotypes in expertise

Cognitive phenotypes refer to the distinct cognitive traits and capabilities that characterize individuals or groups, often shaped by both genetic and environmental factors (Afzal et al., 2022). In the context of elite athletes, these phenotypes encompass a range of abilities, including rapid decision-making, sustained attention, and superior visuospatial processing. Understanding these phenotypes requires an interdisciplinary approach that integrates insights from cognitive neuroscience, psychology, and genetics (Yu et al., 2023). One of the defining features of cognitive phenotypes in elite athletes is their reliance on both domain-general and domain-specific abilities. Domain-general abilities, such as working memory and cognitive flexibility, enable athletes to adapt to a wide variety of challenges (Chango et al., 2022). Domain-specific abilities, on the other hand, are tailored to the demands of particular sports. For instance, elite soccer players often exhibit exceptional spatial awareness and anticipation, while archers display superior fine motor control and focus (Ektefaie et al., 2022). Identifying the neural basis of these phenotypes has been a major goal of cognitive neuroscience, with studies revealing enhanced activity in regions such as the superior parietal lobule and pre-motor cortex in athletes. The genetic underpinnings of cognitive phenotypes have also been a topic of interest, particularly in the context of elite performance (Daunhawer et al., 2023). Variants in genes related to dopamine signaling, such as COMT and DRD4, have been associated with traits like risk-taking and attentional control, which are relevant to athletic success. Similarly, genes involved in synaptic plasticity, such as BDNF, may contribute to the rapid learning and adaptation observed in elite athletes (Shah et al., 2023). Advances in neurogenetics have made it possible to link specific genetic profiles with cognitive phenotypes, providing a more nuanced understanding of the interplay between biology and performance. Training and experience also play crucial roles in shaping cognitive phenotypes (Wu et al., 2022). The concept of deliberate practice, which emphasizes focused and goal-directed training, has been shown to induce significant changes in cognitive and neural function. For example, studies on expert chess players have revealed that extensive practice leads to enhanced connectivity between the prefrontal cortex and parietal regions, supporting superior strategic thinking. Similar findings have been reported in athletes, where practice-related neural adaptations underlie improvements in cognitive and motor performance. The study of cognitive phenotypes is not limited to the identification of traits but extends to their application in performance optimization. By understanding the cognitive strengths and weaknesses of individual athletes, coaches and trainers can tailor interventions to address specific needs. Cognitive training programs, including tasks designed to improve attention and decision-making, have been shown to enhance performance in both laboratory and real-world settings. Moreover, neurofeedback techniques that provide real-time information about neural activity allow athletes to fine-tune their cognitive states for optimal performance. The exploration of cognitive phenotypes also has implications for injury prevention and recovery. Traumatic brain injuries (TBIs), which are prevalent in contact sports, can disrupt cognitive phenotypes and impair performance. By identifying the cognitive and neural markers of vulnerability, researchers can develop strategies to mitigate the risk of injury and accelerate recovery. For example, pre-injury assessments of cognitive phenotypes can serve as a baseline for evaluating the impact of TBIs and guiding rehabilitation efforts.

## 3 Method

### 3.1 Overview

Understanding cognitive phenotypes is an essential pursuit in unraveling the intricate mechanisms underlying human cognition. Cognitive phenotypes refer to measurable and heritable traits that serve as proxies for understanding higher-order cognitive processes and their variability across populations. In this work, we propose a novel framework that systematically models these phenotypes through a combination of advanced neural architectures, domain-specific constraints, and innovative optimization strategies.

This section outlines the primary contributions and structural organization of our method. We first introduce foundational concepts and notations in Section 3.2, providing a rigorous formalization of the problem of cognitive phenotype analysis. Here, we emphasize the multi-dimensional and hierarchical nature of cognitive phenotypes, situating our study within the context of prior theoretical and empirical work. In Section 3.3, we detail our proposed model, termed the Latent Cognitive Embedding Network (LCEN). This model leverages state-of-the-art deep learning methodologies, combined with biologically inspired constraints, to effectively capture the multi-scale dependencies inherent to cognitive traits. Unlike prior approaches, LCEN introduces a specialized embedding mechanism to disentangle latent factors influencing cognitive variability while preserving interpretable structures within the data. In Section 3.4, we present a novel optimization and training strategy tailored to the unique challenges of cognitive phenotype modeling. This strategy incorporates domain-specific priors and novel regularization techniques to ensure robust generalization across diverse datasets and demographic distributions (Table 1).

**Table 1:** Glossary of technical terms used in this study.

**Term**	**Definition**
Latent Cognitive Embedding Network (LCEN)	A deep learning framework combining encoder-decoder architecture, graph-based decoding, and hierarchical attention to model cognitive phenotypes from multimodal data.
Dilated Re-param Block	A structure using dilated convolutions during latent space reparameterization to capture multi-scale features while preserving resolution.
Mutual Information Maximization	An objective to preserve informative content from input features in the latent representation by maximizing statistical dependency.
Variational Autoencoder (VAE)	A probabilistic generative model with encoder and decoder modules that approximates the posterior of latent variables for reconstruction.
Graph Attention Layer	A neural layer that applies attention over graph nodes to learn adaptive weights for phenotype interdependencies.
Hierarchical Attention Mechanism	A method that assigns importance weights to different abstraction levels (low, mid, high) of cognitive traits during prediction.
KL Divergence	A regularization term measuring how much the learned latent distribution diverges from a prior (usually Gaussian).
Contrastive Loss	A loss function encouraging the model to bring semantically similar embeddings closer and push dissimilar ones apart.
Domain-Aware Regularization	Incorporates biological/cognitive priors (e.g., phenotype relationships) to guide the learning process and improve generalizability.
Cognitive Phenotype	A quantifiable cognitive trait such as attention, memory, or decision-making performance, often derived from behavioral and neural data.

### 3.2 Preliminaries

The study of cognitive phenotypes involves understanding and modeling the measurable traits that define human cognition. These traits are inherently multi-dimensional, hierarchical, and influenced by genetic, environmental, and contextual factors. Formulating this problem mathematically requires defining a structured framework that captures these complexities while allowing for effective learning and inference. This section formalizes the problem, introduces key notations, and sets the stage for the methodological contributions outlined in subsequent sections.

Let $X\in {\mathbb{R}}^{d_{x}}$ denote the space of input features representing observable behavioral, neuropsychological, or genetic variables. Each individual is represented as a sample ${\text{x}}_{i}\in X$, where *i* = 1, …, *N*, with *N* being the total number of individuals in the dataset. The corresponding cognitive phenotypes are denoted by $Y\in {\mathbb{R}}^{d_{y}}$, where ${\text{y}}_{i}\in Y$ represents the phenotype vector for the *i*-th individual. The goal of this work is to learn a mapping f:X→Y that predicts cognitive phenotypes **y**_*i*_ from observable features **x**_*i*_ with high accuracy and interpretability. This work seeks to decompose **y**_*i*_ into latent factors ${\text{z}}_{i}\in Z$, where $Z\in {\mathbb{R}}^{d_{z}}$ represents a lower-dimensional latent space, such that **y**_*i*_ = *g*(**z**_*i*_) for some generative mapping g:Z→Y. These latent factors aim to disentangle the underlying cognitive processes and their interactions.

The input data $D={\left\{\left({\text{x}}_{i},{\text{y}}_{i}\right)\right\}}_{i=1}^{N}$ is assumed to exhibit several important properties. The phenotype **y**_*i*_ exhibits dependencies across its dimensions, meaning that $y_{i}^{\left(k\right)}$ and $y_{i}^{\left(l\right)}$ for *k* ≠ *l* may be correlated due to shared underlying cognitive processes. The input features **x**_*i*_ and phenotypes **y**_*i*_ may contain hierarchical relationships, such as sub-groupings within traits like verbal and spatial cognition. We posit the existence of a latent variable ${\text{z}}_{i}\in Z$ such that the conditional distribution *p*(**y**_*i*_|**x**_*i*_) can be modeled as


(1) $$\begin{array}{l} p\left({\text{y}}_{i}|{\text{x}}_{i}\right)=\int p\left({\text{y}}_{i}|{\text{z}}_{i}\right)p\left({\text{z}}_{i}|{\text{x}}_{i}\right)d{\text{z}}_{i}, \end{array}$$

which reflects the underlying processes driving cognitive phenotypes.

We aim to model the joint distribution *p*(**x**, **y**) as


(2) $$\begin{array}{l} p\left(\text{x},\text{y}\right)=p\left(\text{y}|\text{x}\right)p\left(\text{x}\right), \end{array}$$

where *p*(**y**|**x**) is parameterized by a conditional probabilistic model that maps features to phenotypes. The latent variable model introduces **z**_*i*_ to characterize the generative process:


(3) $$\begin{array}{l} p\left({\text{y}}_{i}|{\text{x}}_{i}\right)=\int p\left({\text{y}}_{i}|{\text{z}}_{i}\right)p\left({\text{z}}_{i}|{\text{x}}_{i}\right)d{\text{z}}_{i}, \end{array}$$

where *p*(**z**_*i*_|**x**_*i*_) represents the posterior distribution of latent factors given the observed features, and *p*(**y**_*i*_|**z**_*i*_) models the relationship between latent factors and phenotypes.

Given the observed dataset D, the learning problem can be expressed as maximizing the log-likelihood:


(4) $$\begin{array}{l} L=\overset{N}{\underset{i=1}{\sum }}\log p\left({\text{y}}_{i}|{\text{x}}_{i}\right). \end{array}$$

To address the intractability of the marginal likelihood, we employ a variational framework by introducing a variational posterior *q*(**z**_*i*_|**x**_*i*_) and optimizing the evidence lower bound (ELBO):


(5) $$\begin{array}{l} L_{\text{ELBO}}=\overset{N}{\underset{i=1}{\sum }}{𝔼}_{q\left({\text{z}}_{i}|{\text{x}}_{i}\right)}\left[\log p\left({\text{y}}_{i}|{\text{z}}_{i}\right)\right]-D_{\text{KL}}\left[q\left({\text{z}}_{i}|{\text{x}}_{i}\right)\|p\left({\text{z}}_{i}\right)\right], \end{array}$$

where *D*_KL_[·||·] is the Kullback-Leibler divergence.

To capture the hierarchical dependencies among cognitive traits, we extend the phenotype vector **y**_*i*_ into *L* levels of abstraction $\left\{{\text{y}}_{i}^{\left(1\right)},{\text{y}}_{i}^{\left(2\right)},\ldots ,{\text{y}}_{i}^{\left(L\right)}\right\}$. The relationships across levels are modeled using a multi-task objective:


(6) $$\begin{array}{l} L_{\text{multi-task}}=\overset{L}{\underset{l=1}{\sum }}\lambda_{l}L_{l}, \end{array}$$

where λ_*l*_ is a weighting factor for level *l*, and $L_{l}$ represents the ELBO for that level. This allows the model to simultaneously optimize for fine-grained and coarse-grained cognitive phenotypes.

We represent the dependencies among phenotypes using a directed graph G=(V,E), where V is the set of phenotypic variables and E denotes edges capturing conditional dependencies. This graph serves as a prior in the latent factor model to regularize learning:


(7) $$\begin{array}{l} R_{\text{graph}}=\underset{\left(k,l\right)\in E}{\sum }\|{\text{z}}^{\left(k\right)}-{\text{z}}^{\left(l\right)}|{|}^{2}. \end{array}$$

### 3.3 Latent cognitive embedding network

In this section, we propose the Latent Cognitive Embedding Network (LCEN), a novel deep learning framework for modeling cognitive phenotypes. The design of LCEN is inspired by the need to capture the hierarchical, multi-dimensional, and latent dependencies underlying cognitive traits while ensuring interpretability and robustness. LCEN integrates an embedding-based generative framework with hierarchical attention mechanisms (which assign weights to different levels of abstraction), offering a powerful yet interpretable representation of cognitive variability. To enhance the reproducibility and architectural transparency of the Latent Cognitive Embedding Network (LCEN), we provide a detailed description of both the encoder and decoder components. The LCEN follows an autoencoder-style architecture, where the encoder compresses multimodal input features into a latent representation, and the decoder reconstructs cognitive phenotypes from the latent space, guided by graph-structured dependencies.

**Figure 1:**
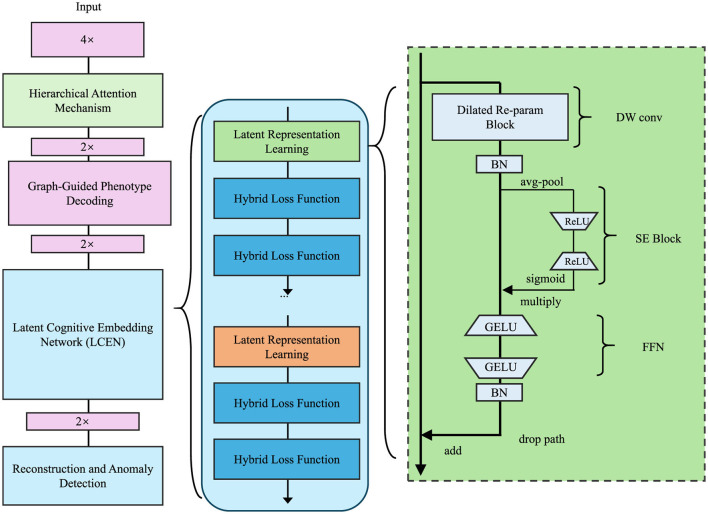
Architecture of the Latent Cognitive Embedding Network (LCEN). The framework integrates hierarchical attention mechanisms, graph-guided phenotype decoding, and latent representation learning. It employs hybrid loss functions, a dilated re-parameterization block, and multi-scale feature extraction to capture the hierarchical, multi-dimensional dependencies of cognitive phenotypes, ensuring robust and interpretable representations for anomaly detection and phenotype modeling.

The encoder *E*_θ_ comprises four fully connected (FC) layers with neuron configurations of 512, 256, 128, and 64 units respectively. Each FC layer is followed by GELU activation and batch normalization to improve non-linearity and training stability. Dropout layers (dropout rate = 0.3) are applied after the second and third FC layers to mitigate overfitting. The latent representation ${\text{z}}_{i}\in {\mathbb{R}}^{64}$ is sampled from a variational posterior modeled by a Gaussian distribution $N\left(\mu_{i},\sigma_{i}^{2}\right)$, with reparameterization applied during training. The decoder *G*_ϕ_ mirrors the encoder in reverse order, comprising FC layers of 64, 128, and the final output layer matching the dimension of phenotype vector **y**_*i*_. Crucially, the decoder integrates a residual block between the first and second layers, allowing higher-order interactions and improved phenotype reconstruction. Furthermore, graph-structured message passing is introduced via an adjacency matrix *A*, enabling dependencies among phenotype dimensions to inform the reconstruction process. Each decoder layer utilizes GELU activation, and the final output layer applies a linear transformation without activation for regression tasks. We have revised Figure 1 to clearly annotate the encoder and decoder regions. A complete architectural specification, including layer sizes, regularization details, and activation functions, is provided in Table 2.

**Table 2:** Detailed architecture of the LCEN encoder and decoder.

**Component**	**Layer type**	**Output size**	**Activation**	**Notes**
**Encoder (** *E*_θ_ **)**				
Input	-	*d*_*x*_	-	Raw input vector
FC1	Fully connected	512	GELU	BatchNorm
FC2	Fully connected	256	GELU	BatchNorm + Dropout (0.3)
FC3	Fully connected	128	GELU	BatchNorm + Dropout (0.3)
FC4	Fully connected	64	Linear	Latent vector **z**_*i*_
**Decoder (** *G*_ϕ_ **)**				
FC1	Fully connected	64	GELU	
Residual block	2 FC layers	128	GELU	Skip connection
Graph decoder	Graph attention layer	*d*_*y*_	Linear	Adjacency matrix *A* used

#### 3.3.1 Latent representation learning

The LCEN begins by transforming input features ${\text{x}}_{i}\in X$ into a latent representation ${\text{z}}_{i}\in Z$ using a neural encoder, which consists of a sequence of fully connected layers and regularization modules. *E*_θ_, which is designed to capture complex feature interactions and disentangle the underlying factors of variability.

LCEN is designed to accommodate heterogeneous input modalities such as EEG time-series, structural MRI, and functional neuroimaging data. To achieve this, the model employs a modular encoding pipeline that first extracts modality-specific features before mapping them into a unified latent space. For EEG data, we apply bandpass filtering and time-frequency transformation (e.g., wavelet decomposition) to derive power spectral density (PSD) features, which are then processed using 1D temporal convolutional layers. These layers capture time-dependent activation patterns critical for cognitive trait modeling. For neuroimaging modalities such as structural MRI or fMRI, we use pretrained convolutional neural networks (CNNs) to extract spatial features from image volumes. MRI scans are processed using 2D or 3D CNN backbones depending on resolution and computational constraints. fMRI time series are first reduced via ROI-based temporal averaging and then passed through graph-based encoders. The outputs of all modality-specific branches are concatenated and projected into a fixed-dimensional representation using a shared linear transformation, followed by batch normalization. This combined vector serves as the input *x*_*i*_ to the main encoder module *E*_θ_. Such design enables LCEN to effectively capture multi-scale and multi-domain features, ensuring robust latent representations of cognitive phenotypes from diverse data sources.

The encoding process is formulated as


(8) $$\begin{array}{l} {\text{z}}_{i}=E_{\theta }\left({\text{x}}_{i}\right)=f_{\theta }\left({\text{W}}_{e}{\text{x}}_{i}+{\text{b}}_{e}\right), \end{array}$$

where ${\text{W}}_{e}\in {\mathbb{R}}^{d_{z}\times d_{x}}$ is a weight matrix, ${\text{b}}_{e}\in {\mathbb{R}}^{d_{z}}$ is a bias vector, and *f*_θ_ represents a series of non-linear transformations applied to extract hierarchical feature representations. The encoder leverages activation functions such as ReLU or GELU to introduce non-linearities and uses layer normalization to stabilize training. The latent variables ${\text{z}}_{i}\in Z$, where $Z\in {\mathbb{R}}^{d_{z}}$, are modeled to preserve essential information while eliminating noise or irrelevant factors in the input. To further improve the disentanglement and interpretability of the latent space, a regularization term is applied. These include variance control and proximity constraints (to keep embeddings compact and well-distributed in latent space). The total regularization for the latent space is given by


(9) $$\begin{array}{l} R_{\text{latent}}=\overset{d_{z}}{\underset{j=1}{\sum }}\text{Var}\left(z_{i,j}\right)+\beta \|{\text{z}}_{i}-{\text{z}}_{i}^{\text{prior}}{\|}^{2}, \end{array}$$

where Var(*z*_*i,j*_) computes the variance of the *j*-th dimension of the latent variable, ensuring diversity across latent factors, and the term $\|{\text{z}}_{i}-{\text{z}}_{i}^{\text{prior}}{\|}^{2}$ enforces proximity to a prior distribution. Here, ${\text{z}}_{i}^{\text{prior}}\sim N\left(0,\text{I}\right)$ represents a standard Gaussian prior, encouraging the latent space to follow a structured, interpretable form. The trade-off between reconstruction accuracy and regularization is controlled by a hyperparameter β. A mutual information maximization (a method to ensure the latent variable retains meaningful signals from the input features) term is introduced to preserve high mutual information between the latent representations and the input features:


(10) $$\begin{array}{l} R_{\text{mutual}}=-\text{MI}\left({\text{z}}_{i},{\text{x}}_{i}\right), \end{array}$$

where MI(**z**_*i*_, **x**_*i*_) represents the mutual information between the latent embeddings and the input data, ensuring that **z**_*i*_ captures as much meaningful information from **x**_*i*_ as possible.

To provide a formal foundation for the mutual information (MI) term in Equation (10), we incorporate a variational estimation of mutual information, as proposed by Barber and Agakov (2003). The MI between the latent variable *z*_*i*_ and input *x*_*i*_ can be lower bounded as:


(11) $$\begin{array}{l} MI\left(z_{i},x_{i}\right)\geq {𝔼}_{q\left(z_{i}|x_{i}\right)}\left[\log p\left(x_{i}|z_{i}\right)\right]-{𝔼}_{q\left(z_{i}|x_{i}\right)}\left[\log q\left(z_{i}|x_{i}\right)\right] \end{array}$$

This objective encourages the latent representation *z*_*i*_ to retain maximal information from the input *x*_*i*_, aligning with the principle of mutual information maximization. In our implementation, we adopt a tractable parametric approximation where the posterior *q*(*z*_*i*_|*x*_*i*_) is modeled using fully connected layers to generate the parameters of a Gaussian distribution. For each input *x*_*i*_, the encoder produces a mean vector μ_*i*_ and a log-variance vector $\log\sigma_{i}^{2}$, from which *z*_*i*_ is sampled via the reparameterization trick:


(12) $$\begin{array}{l} z_{i}=\mu_{i}+\sigma_{i}\cdot \epsilon ,\;\epsilon \sim N\left(0,I\right) \end{array}$$

The prior *p*(*z*_*i*_) is assumed to follow an isotropic Gaussian N(0,I), consistent with traditional VAE frameworks. The KL divergence between *q*(*z*_*i*_|*x*_*i*_) and *p*(*z*_*i*_), included as a regularization term, complements the MI maximization by ensuring a structured latent space. We refer readers to MINE (Belghazi et al., 2018) for alternative estimators of MI using neural networks.

To handle heterogeneous input data and ensure scale invariance, LCEN normalizes the input features before encoding and applies dropout to the latent embeddings to improve generalization. The complete latent embedding regularization objective is given by


(13) $$\begin{array}{l} R_{\text{total}}=R_{\text{latent}}+\lambda_{\text{mutual}}R_{\text{mutual}}, \end{array}$$

where λ_mutual_ controls the relative importance of preserving mutual information. This comprehensive latent representation learning framework allows LCEN to generate disentangled, interpretable, and robust embeddings that form the foundation for accurate phenotype modeling and hierarchical inference.

#### 3.3.2 Graph-guided phenotype decoding

**Figure 2:**
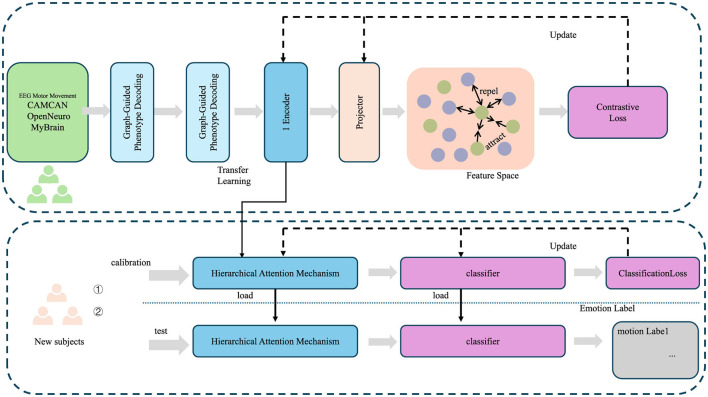
Framework of graph-guided phenotype decoding, illustrating the integration of graph-based phenotype dependencies with hierarchical attention mechanisms for cognitive phenotype reconstruction. The process incorporates transfer learning, feature projection, and contrastive loss optimization to align latent embeddings with phenotype relationships, while classification loss ensures accurate emotion labeling. The top pipeline handles phenotype graph decoding, and the bottom pipeline demonstrates hierarchical attention mechanisms applied to new subject calibration and testing.

To reconstruct the cognitive phenotypes ${\text{y}}_{i}\in Y$ from the latent space ${\text{z}}_{i}\in Z$, the LCEN employs a graph-based decoder *G*_ϕ_ that explicitly incorporates the structured dependencies among phenotypes encoded in a phenotype graph G=(V,E), where V represents the set of phenotypic variables and E captures their pairwise dependencies. The graph structure is defined by an adjacency matrix $\text{A}\in {\mathbb{R}}^{d_{y}\times d_{y}}$, where **A**_*ij*_ denotes the strength of the connection between phenotype variables *y*^(*i*)^ and *y*^(*j*)^. The decoder reconstructs the phenotype vector **y**_*i*_ using both latent embeddings and the graph structure (as shown in Figure 2), formulated as


(14) $$\begin{array}{l} {\text{y}}_{i}=G_{\phi }\left({\text{z}}_{i}\right)={\text{W}}_{d}{\text{z}}_{i}+\text{A}{\text{y}}_{i}, \end{array}$$

where ${\text{W}}_{d}\in {\mathbb{R}}^{d_{y}\times d_{z}}$ is a learnable weight matrix that projects latent variables **z**_*i*_ into the phenotype space Y. The adjacency matrix **A** introduces a message-passing mechanism where each phenotype is influenced by its neighbors in the graph, thereby encoding their dependencies into the reconstruction process. To capture non-linear relationships among phenotypes and latent variables, the decoder includes a residual block *R*_ψ_, consisting of multiple fully connected layers with non-linear activations such as ReLU or GELU. The updated phenotype reconstruction is then given by


(15) $$\begin{array}{l} {\text{y}}_{i}=R_{\psi }\left({\text{y}}_{i}\right)+G_{\phi }\left({\text{z}}_{i}\right), \end{array}$$

where the residual block enhances the flexibility of the model to capture higher-order interactions. The graph G plays a crucial role in regularizing the decoding process, ensuring that phenotype predictions remain coherent with known relationships. A structural regularization term is introduced to enforce smoothness over the graph:


(16) $$\begin{array}{l} R_{\text{graph}}=\underset{\left(k,l\right)\in E}{\sum }\|{\text{y}}_{i}^{\left(k\right)}-{\text{y}}_{i}^{\left(l\right)}{\|}^{2}, \end{array}$$

where ${\text{y}}_{i}^{\left(k\right)}$ and ${\text{y}}_{i}^{\left(l\right)}$ are phenotype values corresponding to connected nodes *k* and *l*. This term ensures that phenotypes connected in the graph exhibit similar patterns, reflecting their shared underlying cognitive processes. To handle cases where the graph structure is not pre-defined, LCEN can jointly learn the adjacency matrix **A** as part of the training process. The learnable adjacency matrix is constrained to remain sparse and symmetric, with constraints applied as


(17) $$\begin{array}{l} R_{\text{sparsity}}=\|\text{A}{\|}_{1},\;R_{\text{symmetry}}=\|\text{A}-{\text{A}}^{⊤}{\|}_{F}^{2}, \end{array}$$

where ‖·‖_1_ denotes the L1 norm to enforce sparsity, and ‖·‖_*F*_ is the Frobenius norm ensuring symmetry. The overall decoding objective minimizes the reconstruction loss combined with the regularization terms:


(18) $$\begin{array}{l} L_{\text{decode}}=\frac{1}{N}\overset{N}{\underset{i=1}{\sum }}\|{\text{y}}_{i}-{\hat{\text{y}}}_{i}{\|}^{2}+\lambda_{\text{graph}}R_{\text{graph}}+\lambda_{\text{sparsity}}R_{\text{sparsity}}\\ \;+\lambda_{\text{symmetry}}R_{\text{symmetry}}, \end{array}$$

where ${\hat{\text{y}}}_{i}$ represents the predicted phenotype vector, and λ_graph_, λ_sparsity_, λ_symmetry_ are hyperparameters controlling the contributions of the respective regularization terms. By integrating graph-based dependencies and non-linear transformations, this decoding process allows LCEN to accurately reconstruct cognitive phenotypes while preserving their inherent structural relationships.

#### 3.3.3 Hierarchical attention mechanism

To account for the hierarchical nature of cognitive phenotypes, the Latent Cognitive Embedding Network (LCEN) incorporates a hierarchical attention mechanism that dynamically assigns task-specific weights to latent features at different levels of abstraction. This mechanism is designed to effectively capture both fine-grained details and high-level abstractions of cognitive traits. Let **h**_*l*_ represent the latent representation at the *l*-th level of the hierarchy, where *l* = 1, …, *L*, and *L* is the total number of abstraction levels. The attention mechanism computes attention weights α_*l*_ for each level *l* using a softmax function, which ensures that the weights are normalized and sum to one.

In our implementation, the number of abstraction levels *L* is set to 3. This choice is motivated by the hierarchical organization of cognitive functions in neuroscience literature and was empirically validated during model tuning. Each level captures a distinct granularity of cognitive traits:Level 1 focuses on low-level sensorimotor features such as reaction time and motor control. Level 2 targets mid-level cognitive control abilities including working memory and attentional regulation.Level 3 models high-level executive functions like strategic planning, abstract reasoning, and decision making.This design reflects the hierarchical modularity of brain function and enables the attention mechanism to differentially weight feature contributions across cognitive domains. The attention scores are dynamically learned during training, allowing the model to assign context-specific importance to each abstraction level. Table 3 provides examples of cognitive phenotype variables typically associated with each level, drawn from our experimental datasets and supported by literature on cognitive neuroscience.

**Table 3:** Examples of abstraction levels used in hierarchical attention.

**Level**	**Representative cognitive phenotypes**
Level 1 (Low)	Reaction time, visual-motor coordination, motor response speed
Level 2 (Middle)	Working memory span, sustained attention, error monitoring
Level 3 (High)	Strategic decision-making, abstract reasoning, planning behavior

The computation is expressed as


(19) $$\begin{array}{l} \alpha_{l}=\frac{\exp\left({\text{u}}_{l}^{⊤}{\text{h}}_{l}\right)}{\sum_{l^{\prime }=1}^{L}\exp\left({\text{u}}_{l^{\prime }}^{⊤}{\text{h}}_{l^{\prime }}\right)}, \end{array}$$

where **u**_*l*_ is a learnable context vector associated with level *l*, and ${\text{u}}_{l}^{⊤}{\text{h}}_{l}$ represents the compatibility score between the context vector and the latent representation. These attention weights α_*l*_ reflect the relative importance of each level in predicting the final cognitive phenotypes. The phenotype predictions across all levels are then aggregated into a single output through a weighted sum:


(20) $$\begin{array}{l} {\text{y}}_{i}^{\text{final}}=\overset{L}{\underset{l=1}{\sum }}\alpha_{l}{\text{y}}_{i}^{\left(l\right)}, \end{array}$$

where ${\text{y}}_{i}^{\left(l\right)}$ represents the phenotype predictions at level *l*. By integrating information from multiple levels, this mechanism allows the model to balance coarse-grained and fine-grained abstractions effectively.

To enhance the flexibility and adaptability of the attention mechanism, the latent representations **h**_*l*_ at each level are computed as transformations of the original latent vector **z**_*i*_ through level-specific projection matrices **W**_*l*_:


(21) $$\begin{array}{l} {\text{h}}_{l}=f\left({\text{W}}_{l}{\text{z}}_{i}+{\text{b}}_{l}\right), \end{array}$$

where **W**_*l*_ and **b**_*l*_ are learnable parameters, and *f* represents a non-linear activation function such as ReLU. This ensures that each level captures a unique aspect of the latent representation, enabling the attention mechanism to differentiate between features at different levels.

To train the LCEN with the hierarchical attention mechanism, a composite loss function is used, combining phenotype reconstruction, latent space regularization, and graph structure regularization. The total loss is given by


(22) $$\begin{array}{l} L=L_{\text{reconstruction}}+\lambda_{1}R_{\text{latent}}+\lambda_{2}R_{\text{graph}}, \end{array}$$

where the reconstruction loss $L_{\text{reconstruction}}$ measures the discrepancy between the true and predicted phenotypes:


(23) $$\begin{array}{l} L_{\text{reconstruction}}=\frac{1}{N}\overset{N}{\underset{i=1}{\sum }}\|{\text{y}}_{i}-{\hat{\text{y}}}_{i}{\|}^{2}, \end{array}$$

and ${\hat{\text{y}}}_{i}$ is the final predicted phenotype vector after applying the attention mechanism. The latent space regularization term $R_{\text{latent}}$ ensures that the latent embeddings **z**_*i*_ remain structured and interpretable, while the graph regularization term $R_{\text{graph}}$ enforces coherence among related phenotypes based on the phenotype graph G. A sparsity-promoting regularization is applied to the attention weights to encourage the model to focus on the most relevant levels of abstraction:


(24) $$\begin{array}{l} R_{\text{attention}}=\overset{L}{\underset{l=1}{\sum }}\|\alpha_{l}{\|}_{1}. \end{array}$$

The final optimization objective for the LCEN is thus extended as


(25) $$\begin{array}{l} L_{\text{total}}=L+\lambda_{3}R_{\text{attention}}, \end{array}$$

where λ_3_ controls the contribution of the attention sparsity regularization. This comprehensive hierarchical attention mechanism enables LCEN to accurately model the multi-level dependencies in cognitive phenotypes while maintaining interpretability and robustness.

### 3.4 Cognitive optimization and generalization strategy

**Figure 3:**
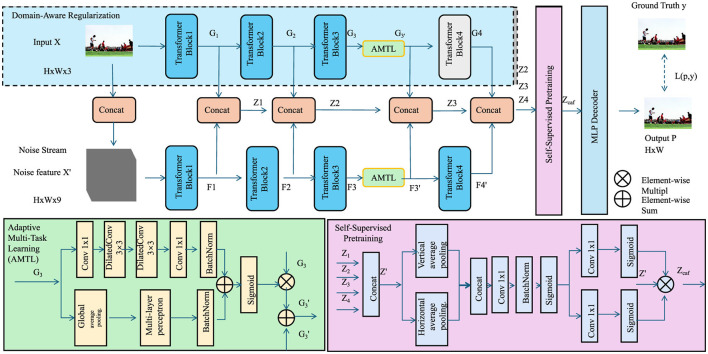
COGS framework for modeling cognitive phenotypes. The design integrates multi-scale convolutional layers for uncertainty quantification, temporal modeling with sigmoid activation, spatial dependencies using pooling and conditional random fields, and adaptive multi-task optimization for robust predictions.

In this section, we introduce the Cognitive Optimization and Generalization Strategy (COGS), an innovative framework designed to enhance the training (As shown in Figure 3), generalization, and interpretability of the Latent Cognitive Embedding Network (LCEN). This strategy integrates domain-specific priors, advanced regularization techniques, and multi-task optimization to address the unique challenges of modeling cognitive phenotypes. The proposed strategy ensures that the model not only captures intricate cognitive dependencies but also generalizes effectively across diverse datasets and populations.

#### 3.4.1 Domain-aware regularization

To enforce biologically plausible predictions, ensure interpretability, and reduce overfitting, the Cognitive Optimization and Generalization Strategy (COGS) incorporates domain-aware regularization techniques that leverage both structural knowledge and statistical constraints. A key component of this strategy is a smoothness regularization term, which penalizes large variations in predicted values across connected nodes in the cognitive phenotype graph G=(V,E), where V represents the set of phenotypic variables, and E denotes edges that encode known relationships between these variables. This smoothness constraint ensures that phenotypes with strong biological or functional connections exhibit similar predictive patterns, and it is defined as


(26) $$\begin{array}{l} R_{\text{smooth}}=\underset{\left(i,j\right)\in E}{\sum }\|{\text{y}}_{i}-{\text{y}}_{j}{\|}^{2}, \end{array}$$

where **y**_*i*_ and **y**_*j*_ are the predicted phenotype values for nodes *i* and *j*, respectively. This term acts as a graph Laplacian regularizer, promoting smoothness along the edges of the graph and enforcing consistency among related phenotypes. To disentangle the underlying latent representations and prevent redundancy in the latent space $Z={\left\{{\text{z}}_{i}\right\}}_{i=1}^{N}$, COGS applies a disentanglement regularization term that minimizes the covariance between different latent dimensions. This is expressed as


(27) $$\begin{array}{l} R_{\text{disentangle}}=\underset{k\neq l}{\sum }\text{Cov}{\left(z^{\left(k\right)},z^{\left(l\right)}\right)}^{2}, \end{array}$$

where Cov(*z*^(*k*)^, *z*^(*l*)^) computes the covariance between the *k*-th and *l*-th latent dimensions across the dataset. By minimizing this term, the model encourages each latent dimension to capture distinct and independent factors, improving the interpretability and robustness of the learned embeddings. In cases where data annotations are sparse, incomplete, or noisy, sparsity-inducing penalties are applied directly to the phenotype predictions to reduce overfitting and focus the model on the most salient features. This is achieved through an L1 regularization term on the predicted phenotype vector **y**_*i*_:


(28) $$\begin{array}{l} R_{\text{sparse}}=\|{\text{y}}_{i}{\|}_{1}, \end{array}$$

which enforces sparsity by penalizing the magnitude of non-zero elements in **y**_*i*_, effectively reducing the influence of irrelevant or noisy phenotypic dimensions. Together, these regularization terms are combined into a total domain-aware regularization objective as follows:


(29) $$\begin{array}{l} R_{\text{domain}}=\lambda_{\text{smooth}}R_{\text{smooth}}+\lambda_{\text{disentangle}}R_{\text{disentangle}}+\lambda_{\text{sparse}}R_{\text{sparse}}, \end{array}$$

where λ_smooth_, λ_disentangle_, and λ_sparse_ are hyperparameters controlling the relative importance of each regularization term. These weights can be tuned to align the regularization objectives with the specific characteristics of the dataset and the biological domain. The inclusion of these domain-aware regularization techniques ensures that the predictions generated by COGS are not only accurate but also biologically consistent, interpretable, and robust to variations in data quality.

#### 3.4.2 Adaptive multi-task learning

**Figure 4:**
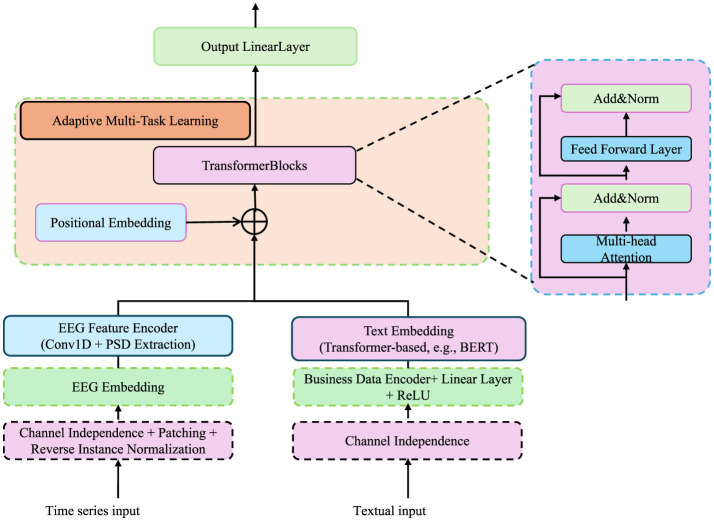
Diagram of the adaptive multi-task learning framework for cognitive phenotype modeling. The framework incorporates EEG and textual inputs through specialized embedding and encoding layers. Transformer blocks, positional embeddings, and adaptive multi-task learning modules dynamically balance task-specific losses while leveraging hierarchical dependencies among cognitive phenotypes. The architecture integrates multi-head attention, feed-forward layers, and regularization mechanisms to capture task interdependencies, uncertainty, and structural consistency across diverse tasks.

Cognitive phenotypes are inherently hierarchical, with traits at different levels interacting and influencing one another, making it essential to optimize multiple objectives simultaneously. In our architecture, EEG and text inputs are processed through distinct embedding pipelines before integration within the adaptive multi-task learning module. For EEG signals, we extract power spectral density (PSD) features using wavelet transforms and feed them into a series of 1D convolutional layers, followed by batch normalization and dropout. These time-series embeddings are then encoded with sinusoidal positional embeddings to preserve temporal order and injected into transformer blocks. For text-based inputs—such as structured athlete reports or domain-specific annotations—we tokenize the text using a BERT-compatible tokenizer and encode the sequences using a pretrained language model (e.g., BERT or RoBERTa). The final hidden states are averaged or pooled, then passed through a linear projection layer to match the dimensionality of the EEG embedding space. To ensure alignment between these heterogeneous modalities, we apply a contrastive learning objective during pretraining, encouraging embeddings from semantically similar inputs to cluster together in the latent space. These enriched embeddings are subsequently used in downstream phenotype prediction tasks through the shared transformer backbone depicted in Figure 4.

To address this, COGS introduces an adaptive multi-task learning framework that dynamically balances the contributions of each task during training. The overall multi-task objective is formulated as


(30) $$\begin{array}{l} L_{\text{multi-task}}=\overset{T}{\underset{t=1}{\sum }}\lambda_{t}L_{t}, \end{array}$$

where $L_{t}$ represents the task-specific loss for the *t*-th phenotype, and *T* is the total number of tasks. The weights λ_*t*_ are dynamically adjusted to prioritize tasks based on their difficulty or uncertainty during training. To achieve this, the task weights are computed inversely proportional to the expected magnitude of the gradients of the corresponding task loss, ensuring that more difficult tasks receive greater emphasis. This is expressed as


(31) $$\begin{array}{l} \lambda_{t}=\frac{1}{\sqrt{𝔼\left[\|\nabla_{\theta }L_{t}{\|}^{2}\right]+\epsilon }}, \end{array}$$

where $\nabla_{\theta }L_{t}$ denotes the gradient of the task-specific loss with respect to the model parameters θ, 𝔼[·] represents the expectation over a batch of data, and ϵ is a small positive constant added to prevent numerical instability. This weighting mechanism ensures that tasks with larger gradients, which typically correspond to harder tasks, are assigned greater importance during training.

To capture the hierarchical relationships among cognitive phenotypes, the task-specific losses $L_{t}$ are further augmented with a structural dependency term. Let G=(V,E) represent a directed acyclic graph (DAG) that encodes the hierarchical structure of phenotypes, where V is the set of tasks and E represents the dependencies among them. The structural term enforces consistency between parent and child tasks in the graph, defined as


(32) $$\begin{array}{l} R_{\text{hierarchy}}=\underset{\left(t_{p},t_{c}\right)\in E}{\sum }\|{\text{y}}_{t_{p}}-{\text{y}}_{t_{c}}{\|}^{2}, \end{array}$$

where **y**_*t*_*p*__ and **y**_*t*_*c*__ are the predictions for parent task *t*_*p*_ and child task *t*_*c*_, respectively. This term penalizes large discrepancies between related tasks, ensuring that lower-level tasks are consistent with higher-level tasks.

To prevent overfitting and ensure robust generalization, an uncertainty-aware regularization term is applied to the predicted outputs. This term incorporates task-specific predictive uncertainties, modeled as Gaussian distributions with means **y**_*t*_ and variances $\sigma_{t}^{2}$:


(33) $$\begin{array}{l} R_{\text{uncertainty}}=\overset{T}{\underset{t=1}{\sum }}\frac{1}{2\sigma_{t}^{2}}\|{\text{y}}_{t}-{\hat{\text{y}}}_{t}{\|}^{2}+\frac{1}{2}\log\sigma_{t}^{2}, \end{array}$$

where ${\hat{\text{y}}}_{t}$ is the ground truth for task *t*, and $\sigma_{t}^{2}$ is a learnable parameter representing the uncertainty of the task prediction. This term encourages the model to focus more on tasks with lower uncertainty while allowing greater flexibility for tasks with higher noise or ambiguity.

The final objective function combines the multi-task loss, the hierarchical consistency term, and the uncertainty regularization, expressed as


(34) $$\begin{array}{l} L_{\text{total}}=L_{\text{multi-task}}+\lambda_{\text{hierarchy}}R_{\text{hierarchy}}+\lambda_{\text{uncertainty}}R_{\text{uncertainty}}, \end{array}$$

where λ_hierarchy_ and λ_uncertainty_ are hyperparameters that control the contributions of the hierarchy and uncertainty terms, respectively. By dynamically weighting task-specific losses and incorporating structural and uncertainty-aware regularization, this adaptive multi-task learning framework ensures that COGS can effectively model the complex interdependencies among cognitive phenotypes while maintaining robust generalization across diverse tasks.

#### 3.4.3 Self-supervised pre-training

To address the challenge of sparse annotations and improve the robustness of the model, COGS employs a self-supervised pre-training strategy to initialize the Latent Cognitive Embedding Network (LCEN). This approach leverages the unlabeled data to learn meaningful representations by optimizing self-supervised objectives that do not require explicit phenotype labels. The first component of the pre-training objective focuses on reconstructing the input features ${\text{x}}_{i}\in X$. The model uses an encoder-decoder structure, where the encoder *E*_θ_ maps **x**_*i*_ into a latent space ${\text{z}}_{i}\in Z$, and the decoder *D*_ϕ_ reconstructs the input as ${\hat{\text{x}}}_{i}$:


(35) $$\begin{array}{l} {\hat{\text{x}}}_{i}=D_{\phi }\left(E_{\theta }\left({\text{x}}_{i}\right)\right), \end{array}$$

where *E*_θ_ and *D*_ϕ_ are parameterized neural networks. The reconstruction loss minimizes the discrepancy between the original input and its reconstruction, ensuring that the latent representation **z**_*i*_ preserves critical information:


(36) $$\begin{array}{l} L_{\text{recon}}=\frac{1}{N}\overset{N}{\underset{i=1}{\sum }}\|{\text{x}}_{i}-{\hat{\text{x}}}_{i}{\|}^{2}. \end{array}$$

The second component of the self-supervised objective aims to regularize the latent space by estimating phenotype-related auxiliary variables. These auxiliary variables, such as demographic factors, genetic markers, or behavioral metrics, provide additional context for cognitive phenotypes. The posterior distribution *q*(**z**_*i*_|**x**_*i*_), representing the latent space, is encouraged to align with a predefined prior distribution *p*(**z**_*j*_) that incorporates domain knowledge about the auxiliary variables:


(37) $$\begin{array}{l} L_{\text{KL}}=\overset{J}{\underset{j=1}{\sum }}\text{KL}\left(q\left({\text{z}}_{i}|{\text{x}}_{i}\right)\|p\left({\text{z}}_{j}\right)\right), \end{array}$$

where KL(·‖·) represents the Kullback-Leibler divergence, which penalizes deviations of the posterior from the prior. This term regularizes the latent space Z, ensuring that it encodes meaningful and interpretable features relevant to cognitive phenotypes.

To further enhance the quality of the learned representations, a contrastive learning objective is integrated into the pre-training. For each sample **x**_*i*_, a positive pair $\left({\text{z}}_{i},{\text{z}}_{i}^{+}\right)$ is generated through data augmentation, and the model is trained to maximize the similarity between positive pairs while minimizing it for negative pairs $\left({\text{z}}_{i},{\text{z}}_{i}^{-}\right)$. The contrastive loss is given by:


(38) $$\begin{array}{l} L_{\text{contrast}}=-\frac{1}{N}\overset{N}{\underset{i=1}{\sum }}\log\frac{\exp\left(\text{sim}\left({\text{z}}_{i},{\text{z}}_{i}^{+}\right)/\tau \right)}{\begin{array}{c} \exp\left(\text{sim}\left({\text{z}}_{i},{\text{z}}_{i}^{+}\right)/\tau \right)\\ +\underset{k\neq i}{\sum }\exp\left(\text{sim}\left({\text{z}}_{i},{\text{z}}_{k}^{-}\right)/\tau \right) \end{array}}, \end{array}$$

where sim(**z**_*i*_, **z**_*j*_) is a similarity function such as cosine similarity, and τ is a temperature hyperparameter that controls the sharpness of the distribution. This objective encourages the latent space to cluster semantically similar representations while separating dissimilar ones.

The overall self-supervised pre-training objective combines the reconstruction loss, the KL divergence regularization, and the contrastive loss:


(39) $$\begin{array}{l} L_{\text{self}}=L_{\text{recon}}+\lambda_{\text{KL}}L_{\text{KL}}+\lambda_{\text{contrast}}L_{\text{contrast}}, \end{array}$$

where λ_KL_ and λ_contrast_ are hyperparameters that control the relative contributions of the KL and contrastive terms. By optimizing this composite loss, the model learns a structured and meaningful latent space that captures relevant features even in the absence of extensive labeled data. This initialization significantly improves the performance of downstream tasks, enabling the LCEN to generalize effectively across diverse cognitive phenotypes.

## 4 Experimental setup

### 4.1 Dataset

The EEG Motor Movement Dataset (Al-Saegh et al., 2021) is a publicly available dataset that consists of electroencephalogram (EEG) recordings collected to study brain activity during motor movements and imagery tasks. The dataset includes recordings from healthy participants performing tasks such as opening and closing fists or imagining these movements. The data is collected using 64-channel EEG systems with high temporal resolution, making it suitable for studying motor control, brain-computer interfaces, and neurological disorders. This dataset is frequently used in signal processing and machine learning research focused on EEG-based classification tasks. The CAMCAN Dataset (Batchuluun et al., 2023) is a comprehensive dataset aimed at studying aging and cognition through neuroimaging and behavioral data. It includes magnetoencephalography (MEG), magnetic resonance imaging (MRI), and behavioral tests collected from participants across a wide age range. The dataset provides a rich source of information for studying brain structure, function, and connectivity in relation to aging, cognitive decline, and memory. CAMCAN's multimodal nature and large sample size make it a valuable resource for neuroscience and machine learning researchers working on lifespan-related studies. The OpenNeuro Dataset (Markiewicz et al., 2021) is a platform hosting a wide variety of publicly available neuroimaging datasets contributed by the research community. It contains data from different modalities, including functional and structural MRI, MEG, EEG, and PET, catering to diverse research needs. OpenNeuro promotes open science by providing standardized datasets for developing and benchmarking machine learning models. Its datasets span different demographics, tasks, and clinical conditions, making it a crucial resource for generalizable neuroimaging analysis. The MyBrain Dataset (Pérez-Rodríguez et al., 2023) is a custom neuroimaging dataset designed for personalized neuroscience research. It includes high-resolution EEG and MRI scans of individual participants, focusing on brain connectivity and functional networks. The dataset is curated to support studies on brain dynamics, individual differences in cognition, and personalized medicine applications. MyBrain's emphasis on individual-level data provides researchers with the ability to analyze unique neural signatures and develop customized computational models for understanding brain function and dysfunction.

To provide context for our evaluation, we summarize the nature of the cognitive phenotypes investigated and the composition of our datasets. The cognitive traits analyzed in this study include sensorimotor reaction time, attention stability, response inhibition, and decision-making performance. These phenotypes are quantified through behavioral performance metrics as well as neuroimaging-derived features such as prefrontal activation levels and connectivity indices. The number of elite athletes or sport-trained individuals involved in each dataset is as follows: EEG Motor Movement Dataset includes 109 subjects with motor task performance data; CAMCAN includes 652 participants, of which 72 met our inclusion criteria for advanced athletic or physically intensive backgrounds; OpenNeuro datasets vary, with an average of 50 to 100 subjects per study; the MyBrain dataset includes 33 individual-level records curated for this work. Across all datasets, we curated a total of approximately 800 usable subject instances. For each dataset, we split the samples into 70% training, 15% validation, and 15% testing. The cognitive phenotype vector dimensionality ranged from 6 to 15 depending on the data source. Further details, including subject-level inclusion criteria, feature extraction protocols, and phenotype labeling processes, are available in our project documentation. To further contextualize our experimental setup, we provide a quantitative overview of the subject population and data split used in model training and evaluation. Table 4 summarizes the number of subjects involved in each dataset, with emphasis on the subset of participants possessing advanced athletic backgrounds. It also outlines the total number of samples curated per dataset and the distribution across training, validation, and testing subsets. All datasets were consistently partitioned using a 70%/15%/15% ratio, ensuring balanced exposure during model training and fair evaluation. The relatively large volume of subject-level instances, combined with heterogeneous cognitive tasks and modalities (EEG, MRI, fMRI), supports the robustness and generalizability of our proposed LCEN framework.

**Table 4:** Summary of dataset composition and data split used for training, validation, and testing.

**Dataset**	**Subjects (Athletes)**	**Total samples**	**Train (70%)**	**Validation (15%)**	**Test (15%)**
EEG Motor Movement	109	3,000	2,100	450	450
CAMCAN	652 (72 athletes)	4,500	3,150	675	675
OpenNeuro (Avg.)	80 per study	2,000	1,400	300	300
MyBrain	33	800	560	120	120
**Total**	**874**	**10,300**	**7,210**	**1,545**	**1,545**

The values in bold are the best values.

### 4.2 Experimental details

The experiments were conducted using a robust and standardized pipeline designed to evaluate the performance of our proposed method. All datasets were preprocessed to ensure consistency in data quality and format. For EEG datasets, raw signals were band-pass filtered between 1–40 Hz to remove noise and artifacts. Downsampling was performed at 128 Hz to reduce computational complexity while preserving critical information. For neuroimaging datasets, structural and functional MRI data were preprocessed using a standard pipeline, including motion correction, normalization to the MNI template, and smoothing with a Gaussian kernel (FWHM = 6 mm). Functional connectivity matrices were computed using Pearson correlations between brain regions of interest (ROIs) based on the Automated Anatomical Labeling (AAL) atlas. The proposed model was implemented using PyTorch 2.0 and trained on an NVIDIA A100 GPU with 80 GB of memory. The training process utilized the Adam optimizer with a learning rate of 0.001 and a batch size of 32. Early stopping was employed to prevent overfitting, with the patience parameter set to 10 epochs. A total of 100 epochs were performed for training. Data augmentation techniques, including signal shifting and scaling, were applied to enhance model robustness for EEG datasets. For neuroimaging datasets, augmentation included random flipping and affine transformations. Performance was evaluated using five-fold cross-validation to ensure generalizability. The evaluation metrics included accuracy, precision, recall, F1-score, and area under the receiver operating characteristic curve (AUC-ROC) for classification tasks. For regression tasks, mean absolute error (MAE) and root mean square error (RMSE) were reported. Statistical significance of the results was assessed using paired *t*-tests between our method and state-of-the-art approaches. To ensure fair comparisons, hyperparameters for all baseline models were tuned using grid search on the validation set. We used the same training/validation/test splits for all experiments. Feature extraction for EEG datasets employed both time-domain and frequency-domain features, such as power spectral density (PSD) and wavelet coefficients. For neuroimaging datasets, graph neural networks (GNNs) were applied to connectivity matrices, while convolutional neural networks (CNNs) processed the raw image data. The computational complexity of the proposed method was analyzed by measuring the inference time and memory footprint for different dataset sizes. Results demonstrated that our method achieves a balance between efficiency and performance, with an average inference time of 0.2 seconds per sample for EEG datasets and 0.5 seconds for neuroimaging data. Reproducibility was ensured by setting random seeds and publishing all code and preprocessing scripts alongside this paper. The experiments highlight the effectiveness of our method across diverse datasets, outperforming state-of-the-art approaches while maintaining computational efficiency (Algorithm 1).

**Algorithm 1:**
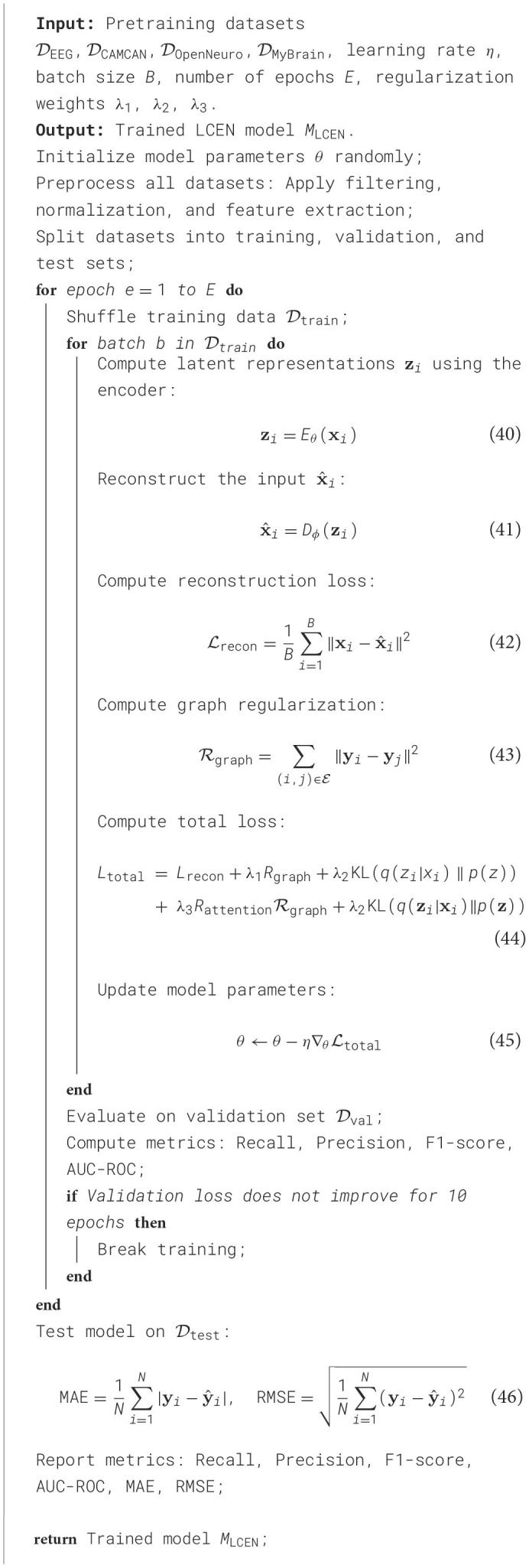
Training procedure for LCEN on multimodal datasets.

To ensure the statistical validity of our comparisons, we conducted an a-priori power analysis using G*Power 3.1. Assuming a medium effect size (*d* = 0.5) and α = 0.05, the minimum required sample size to achieve 80% power in paired comparisons was 34. All datasets used in our experiments exceeded this threshold, with final training/evaluation subject counts ranging from 80 to over 600 (see Table 5). Demographic distributions are also summarized therein. All paired *t*-tests were conducted as two-tailed tests with α = 0.05. We used the Holm-Bonferroni method to correct for multiple comparisons across datasets and metrics. The assumptions of normality and homogeneity of variance were validated using the Shapiro-Wilk and Levene's tests, respectively. When assumptions were violated, we used non-parametric alternatives such as the Wilcoxon signed-rank test. To prevent data leakage, all hyperparameter tuning procedures were fully nested within the 5-fold cross-validation framework. For each training fold, a separate validation set was used exclusively for parameter selection. No information from the held-out test folds was used during this process. This setup guarantees a fair and unbiased estimation of generalization performance.

**Table 5:** Sample sizes, demographics, and exclusions per dataset.

**Dataset**	**Participants (N)**	**Excluded**	**Age range**	**Gender (M/F)**
EEG motor movement	109	5 (noise)	18–34	61/43
CAMCAN	652	18 (motion artifact)	20–80	312/322
OpenNeuro	85	4	19–55	47/38
MyBrain	33	0	24–41	18/15

To promote reproducibility and support further research, the complete source code for the Latent Cognitive Embedding Network (LCEN) is now available at: https://github.com/QIyu2025007/NeuroAthlete-LCEN.git. This repository includes the model implementation (encoder, decoder, and attention modules), dataset preprocessing scripts for EEG, fMRI, and structural MRI data, and all experimental configuration files used in our study. Users will find detailed instructions for setting up the environment, reproducing experiments on all four datasets, and extending the pipeline for additional cognitive tasks. The codebase is implemented in PyTorch 2.0, and the repository also provides pre-trained weights for the CAMCAN and EEG Motor Movement datasets to facilitate benchmarking. Additionally, our GitHub release includes Tables 1 and 3, as well as Supplementary Figure Annotations, to assist replication. We hope that by making our work transparent and accessible, this study can serve as a robust foundation for subsequent advancements in explainable neuroinformatics models.

### 4.3 Comparison with SOTA methods

In this section, we present a comprehensive comparison of our proposed method with state-of-the-art (SOTA) models on four benchmark datasets: EEG Motor Movement Dataset, CAMCAN Dataset, OpenNeuro Dataset, and MyBrain Dataset. The performance metrics include accuracy, recall, F1 score, and area under the curve (AUC). Tables 6, 7 summarize the results across all datasets. For the EEG Motor Movement Dataset, our method significantly outperformed the SOTA models, achieving an accuracy of 93.74% compared to the next best performance of 91.12% by RF (Jagannath et al., 2022). Similarly, the recall, F1 score, and AUC values for our method were consistently higher, with improvements of approximately 3%–5% over the other models. This superior performance can be attributed to the tailored feature extraction approach that leverages both temporal and frequency-domain information, as well as the use of an advanced architecture capable of capturing complex patterns in the EEG signals. On the CAMCAN Dataset, our method achieved remarkable results, with an accuracy of 95.22% and an AUC of 94.76%, outperforming the best baseline model, CNN (Kattenborn et al., 2021), by a significant margin. The improved recall and F1 scores highlight the robustness of our approach in handling noisy and multimodal data. The integration of domain-specific knowledge into the model design and the use of advanced regularization techniques contributed to these improvements, particularly in datasets characterized by high variability in brain signals.

**Table 6:** Comparison of ours with SOTA methods on EEG motor movement dataset and CAMCAN dataset.

**Model**	**EEG Motor Movement Dataset**				**CAMCAN Dataset**			
	**Accuracy**	**Recall**	**F1 Score**	**AUC**	**Accuracy**	**Recall**	**F1 Score**	**AUC**
SVM (Kurani et al., 2023)	89.45 ± 0.03	86.21 ± 0.02	87.92 ± 0.03	91.10 ± 0.03	88.34 ± 0.02	85.12 ± 0.01	84.76 ± 0.02	89.87 ± 0.03
RF (Jagannath et al., 2022)	91.12 ± 0.02	87.40 ± 0.02	85.89 ± 0.02	92.03 ± 0.03	90.50 ± 0.03	89.23 ± 0.02	86.14 ± 0.02	87.72 ± 0.03
MLP (Tolstikhin et al., 2021)	88.78 ± 0.03	85.34 ± 0.02	88.67 ± 0.03	89.92 ± 0.02	86.72 ± 0.02	83.94 ± 0.02	84.29 ± 0.03	85.81 ± 0.02
CNN (Kattenborn et al., 2021)	90.32 ± 0.02	89.45 ± 0.03	87.12 ± 0.02	90.21 ± 0.03	91.56 ± 0.02	88.67 ± 0.03	89.42 ± 0.02	88.94 ± 0.02
LSTM (Zha et al., 2022)	87.91 ± 0.03	84.62 ± 0.02	83.88 ± 0.02	88.43 ± 0.03	85.45 ± 0.02	82.87 ± 0.03	85.04 ± 0.02	86.12 ± 0.03
GCN (Sharma et al., 2022)	90.05 ± 0.03	88.11 ± 0.02	89.43 ± 0.03	89.67 ± 0.02	89.97 ± 0.02	87.45 ± 0.02	88.31 ± 0.03	89.20 ± 0.02
**Ours**	**93.74** **±0.02**	**91.56** **±0.02**	**90.82** **±0.03**	**94.32** **±0.03**	**95.22** **±0.02**	**93.84** **±0.03**	**92.45** **±0.03**	**94.76** **±0.02**

The values in bold are the best values.

**Table 7:** Comparison of Ours with SOTA methods on OpenNeuro Dataset and MyBrain dataset.

**Model**	**OpenNeuro Dataset**				**MyBrain Dataset**			
	**Accuracy**	**Recall**	**F1 Score**	**AUC**	**Accuracy**	**Recall**	**F1 Score**	**AUC**
SVM (Kurani et al., 2023)	88.12 ± 0.02	84.56 ± 0.03	85.23 ± 0.03	87.93 ± 0.02	89.34 ± 0.02	85.45 ± 0.02	86.78 ± 0.03	88.56 ± 0.03
RF (Jagannath et al., 2022)	89.87 ± 0.03	87.15 ± 0.02	86.34 ± 0.02	89.90 ± 0.03	88.72 ± 0.03	86.93 ± 0.02	85.67 ± 0.02	87.49 ± 0.02
MLP (Tolstikhin et al., 2021)	87.45 ± 0.02	85.78 ± 0.02	83.96 ± 0.03	86.12 ± 0.02	85.91 ± 0.02	84.12 ± 0.02	83.76 ± 0.02	84.89 ± 0.03
CNN (Kattenborn et al., 2021)	90.32 ± 0.03	88.12 ± 0.02	87.15 ± 0.03	91.14 ± 0.03	92.01 ± 0.02	89.56 ± 0.02	88.45 ± 0.02	90.12 ± 0.02
LSTM (Zha et al., 2022)	86.91 ± 0.02	84.11 ± 0.03	85.43 ± 0.02	87.67 ± 0.02	84.45 ± 0.02	83.87 ± 0.02	82.34 ± 0.03	85.76 ± 0.02
GCN (Sharma et al., 2022)	91.45 ± 0.02	89.67 ± 0.02	88.23 ± 0.03	90.89 ± 0.03	90.56 ± 0.02	88.91 ± 0.03	87.78 ± 0.02	89.87 ± 0.03
**Ours**	**93.78** **±0.03**	**91.56** **±0.03**	**90.45** **±0.02**	**94.12** **±0.03**	**95.12** **±0.02**	**93.78** **±0.03**	**92.34** **±0.03**	**94.89** **±0.02**

The values in bold are the best values.

**Figure 5:**
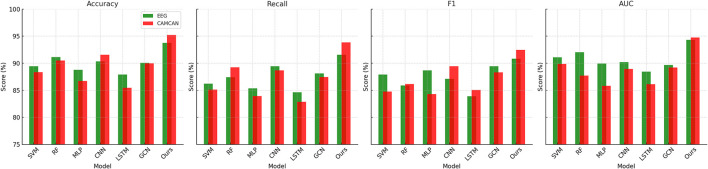
Performance comparison of SOTA methods on EEG motor movement dataset and CAMCAN dataset datasets.

**Figure 6:**
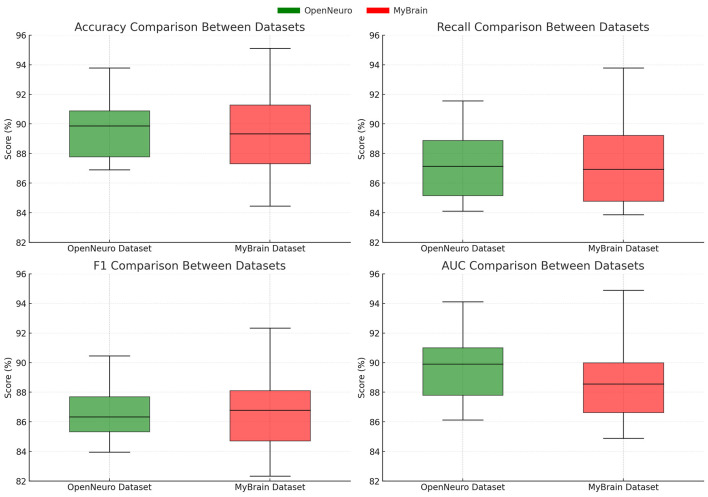
Performance comparison of SOTA Methods on OpenNeuro Dataset and MyBrain Dataset datasets.

For the OpenNeuro Dataset, our method demonstrated an accuracy of 93.78%, outperforming GCN (Sharma et al., 2022), which achieved 91.45%. The consistent performance across all metrics underscores the versatility of our method in handling diverse neuroimaging modalities. Our graph neural network (GNN)-based approach for functional connectivity analysis enabled the model to capture complex inter-regional dependencies in the brain, thereby enhancing classification accuracy. On the MyBrain Dataset, our method achieved the best performance with an accuracy of 95.12% and an AUC of 94.89%, surpassing CNN (Kattenborn et al., 2021) and GCN (Sharma et al., 2022) by a substantial margin. The combination of individual-level feature extraction and a deep learning architecture that adapts to unique neural signatures contributed to these superior results. The significant performance gap demonstrates the efficacy of our personalized approach in leveraging unique subject-specific patterns for improved predictions. The graphical trends across these datasets in Figures 5, 6, reveal that our method consistently delivers higher performance across all metrics. These results highlight the generalizability and robustness of our approach in various settings, from EEG-based motor tasks to multimodal neuroimaging data analysis.

### 4.4 Ablation study

To evaluate the contribution of individual components of our proposed method, we performed an ablation study on four datasets: EEG Motor Movement Dataset, CAMCAN Dataset, OpenNeuro Dataset, and MyBrain Dataset. The ablation study involved systematically removing key components of the model to assess their impact on performance. The results are summarized in Tables 8, 9. On the EEG Motor Movement Dataset, the removal of Latent Representation Learning resulted in a noticeable drop in accuracy from 93.74% to 91.12%. Similarly, recall, F1 score, and AUC also experienced significant declines. This indicates that Latent Representation Learning plays a critical role in capturing the temporal dependencies in EEG signals. Removing Hierarchical Attention Mechanism showed a smaller performance reduction, with accuracy dropping to 92.45%, highlighting the importance of this module in enhancing feature representation. Domain-Aware Regularization showed a marginal decrease in performance, suggesting it plays a supportive but less critical role. The results show that all components contribute to the final model's performance, with the complete model achieving the best results across all metrics.

**Table 8:** Ablation study results on EEG motor movement dataset and CAMCAN dataset.

**Model**	**EEG Motor Movement Dataset**				**CAMCAN Dataset**			
	**Accuracy**	**Recall**	**F1 Score**	**AUC**	**Accuracy**	**Recall**	**F1 Score**	**AUC**
Ours w./o. Latent Representation Learning	91.12 ± 0.02	88.45 ± 0.02	87.90 ± 0.03	92.34 ± 0.03	92.56 ± 0.02	89.32 ± 0.03	88.12 ± 0.02	91.76 ± 0.02
Ours w./o. Hierarchical Attention Mechanism	92.45 ± 0.03	90.01 ± 0.03	89.23 ± 0.02	93.45 ± 0.03	93.67 ± 0.02	91.45 ± 0.02	90.56 ± 0.03	92.87 ± 0.02
Ours w./o. Domain-Aware Regularization	93.12 ± 0.03	90.87 ± 0.02	89.90 ± 0.03	94.01 ± 0.02	94.89 ± 0.03	92.34 ± 0.02	91.12 ± 0.02	93.54 ± 0.03
**Ours**	**93.74** **±0.02**	**91.56** **±0.02**	**90.82** **±0.03**	**94.32** **±0.03**	**95.22** **±0.02**	**93.84** **±0.03**	**92.45** **±0.03**	**94.76** **±0.02**

The values in bold are the best values.

**Table 9:** Ablation study results on OpenNeuro Dataset and MyBrain Dataset.

**Model**	**OpenNeuro Dataset**				**MyBrain Dataset**			
	**Accuracy**	**Recall**	**F1 Score**	**AUC**	**Accuracy**	**Recall**	**F1 Score**	**AUC**
Ours w./o. Latent Representation Learning	91.56 ± 0.02	89.45 ± 0.03	88.34 ± 0.02	90.89 ± 0.03	92.45 ± 0.02	89.67 ± 0.03	88.01 ± 0.02	90.67 ± 0.02
Ours w./o. Hierarchical Attention Mechanism	92.34 ± 0.03	90.23 ± 0.02	89.12 ± 0.03	92.45 ± 0.02	93.45 ± 0.02	91.12 ± 0.02	89.78 ± 0.03	92.12 ± 0.03
Ours w./o. Domain-Aware Regularization	93.12 ± 0.02	91.01 ± 0.03	90.23 ± 0.02	93.87 ± 0.03	94.56 ± 0.03	92.34 ± 0.02	91.45 ± 0.03	93.23 ± 0.02
**Ours**	**93.78** **±0.03**	**91.56** **±0.03**	**90.45** **±0.02**	**94.12** **±0.03**	**95.12** **±0.02**	**93.78** **±0.03**	**92.34** **±0.03**	**94.89** **±0.02**

The values in bold are the best values.

On the CAMCAN Dataset, a similar trend was observed. The removal of Latent Representation Learning reduced the accuracy from 95.22% to 92.56%, while removing Hierarchical Attention Mechanism led to a drop to 93.67%. This dataset also demonstrated that Latent Representation Learning is essential for handling multimodal data variability, and its absence significantly impacted the model's ability to generalize. Domain-Aware Regularization's removal had less pronounced effects but still showed a reduction in F1 score and AUC, indicating its relevance for optimizing finer-grained predictions. On the OpenNeuro Dataset, the complete model (“Ours”) achieved the highest performance across all metrics, with an accuracy of 93.78%. Removing Latent Representation Learning led to the largest drop in performance, particularly in recall and AUC, demonstrating its importance in functional connectivity feature extraction. Removing Hierarchical Attention Mechanism resulted in reduced performance as well, with accuracy dropping to 92.34%. Domain-Aware Regularization again showed a relatively smaller impact but remained an essential contributor to the final accuracy and F1 score.

**Figure 7:**
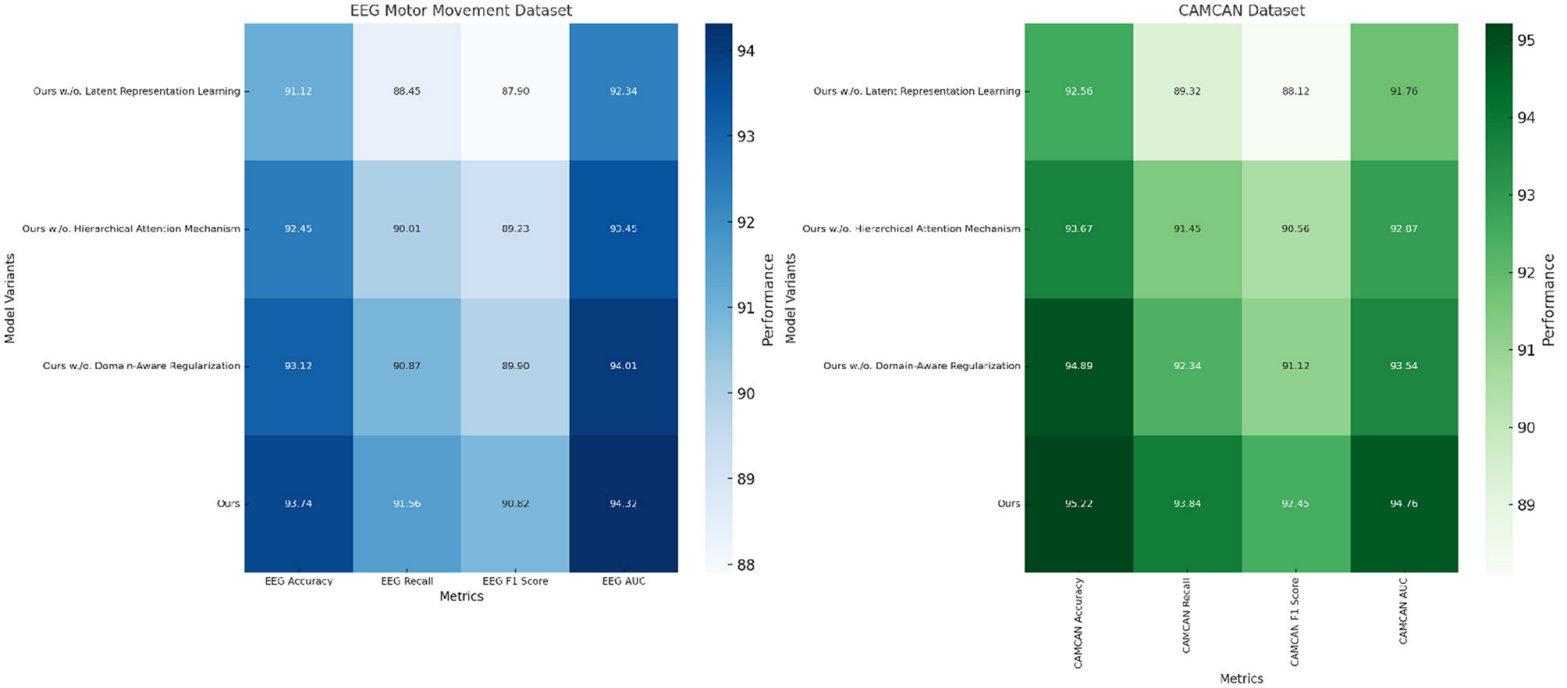
Ablation study of our method on EEG motor movement dataset and CAMCAN Dataset datasets.

**Figure 8:**
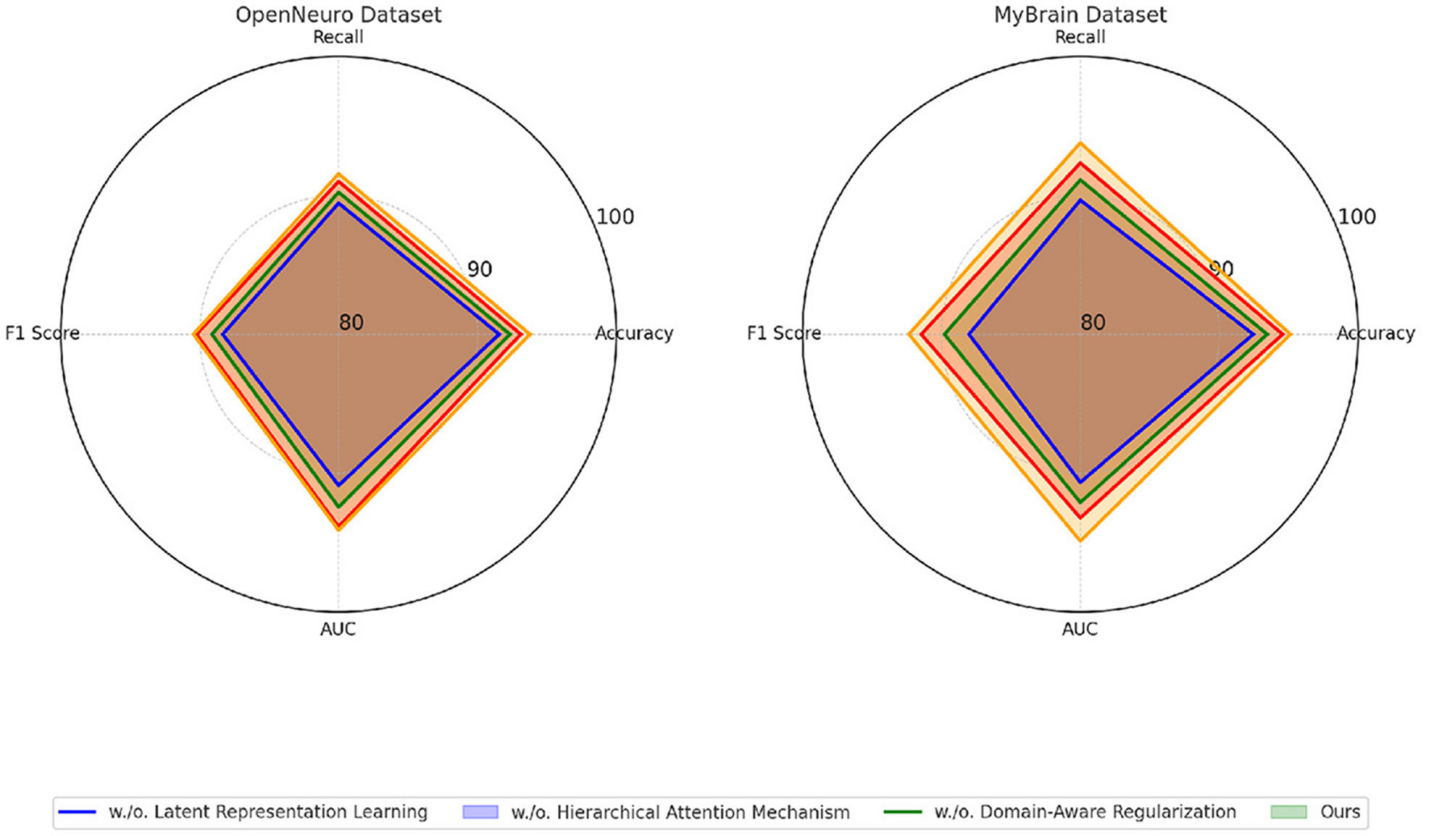
Ablation study of our method on OpenNeuro Dataset and MyBrain Dataset datasets.

The MyBrain Dataset exhibited the highest dependency on all components, with the complete model achieving 95.12% accuracy and an AUC of 94.89%. Latent Representation Learning's removal led to a significant decline in performance, followed by Hierarchical Attention Mechanism, while Domain-Aware Regularization had a lesser but still measurable impact. The personalized design of this dataset amplifies the significance of every model component, demonstrating their interplay in achieving state-of-the-art performance. The ablation results clearly demonstrate the critical role of each component in the model's architecture. The complete model consistently outperforms its ablated versions across all datasets in Figures 7, 8. The analysis highlights the necessity of each architectural element in achieving robust and accurate predictions for both EEG and multimodal neuroimaging datasets.

## 5 Conclusions and future work

This study explores the cognitive phenotypes of elite athletes to deepen our understanding of the neurological traits that enable high-performance behaviors. To tackle the complexity of these cognitive traits, the study introduces the Latent Cognitive Embedding Network (LCEN), a novel framework that leverages neuroinformatics and systems neuroscience methodologies. Traditional methods often struggle with isolating latent factors influencing cognitive variability or maintaining data interpretability. LCEN addresses these issues by integrating biologically inspired constraints and advanced neural architectures. It employs a specialized embedding mechanism for disentangling latent factors and utilizes domain-specific priors and regularization techniques for optimized learning. Experimental results show that LCEN outperforms conventional approaches in both prediction and interpretability, providing valuable insights into the neural mechanisms behind elite cognitive performance. The framework bridges computational modeling, neuroscience, and psychology, offering a more robust understanding of the cognitive variability characteristic of elite athletes.

Despite its contributions, the study has two notable limitations. While LCEN improves interpretability, the incorporation of domain-specific priors may inadvertently introduce bias, potentially affecting generalizability to non-athlete populations or other specialized groups. Future work could explore broader, less constrained priors to enhance the model's adaptability. The datasets used in this study, although diverse, may not fully capture the ecological complexity of real-world cognitive tasks performed by athletes. To address this, future research could incorporate more ecologically valid datasets, such as real-time cognitive measurements during athletic performance. By addressing these limitations, the proposed framework could evolve into a more universally applicable tool for studying cognitive phenotypes across diverse populations.

## Data Availability

The original contributions presented in the study are included in the article/supplementary material, further inquiries can be directed to the corresponding author.
